# Validity and Reliability of a Test Battery to Assess Change of Directions with Ball Dribbling in Para-footballers with Cerebral Palsy

**DOI:** 10.3390/brainsci10020074

**Published:** 2020-01-31

**Authors:** Lucas Felippe Daniel, Raúl Reina, José Irineu Gorla, Tânia Bastos, Alba Roldan

**Affiliations:** 1 Centre of Research, Education, Innovation and Intervention in Sport (CIFI2D), Faculty of Sport, University of Porto, 4200-450 Porto, Portugal; lfadrj@gmail.com (L.F.D.); tbastos@fade.up.pt (T.B.); 2 Sport Research Centre, Department of Sport Sciences, Miguel Hernández University, 03202 Elche, Spain; aroldan@umh.es; 3 International Federation of Cerebral Palsy Football, 6521 KR Nijmegen, The Netherlands; 4 Laboratory of Physical Education in Adapted Sport and Exercise, Department of Studies in Adapted Physical Education, Faculty of Physical Education, University of Campinas, Campinas 13083-851, Brazil; jigorla@uol.com.br

**Keywords:** brain injury, Paralympic, classification, change of direction

## Abstract

The purpose of this study was to evaluate the content and construct validity and between-sessions reliability of four agility tests requiring ball dribbling in football players with cerebral palsy (CP) with implications for classification and training. A sample of 35 football players with CP from three different countries took part in the study. They performed four tests in two sessions 72 h apart: i) 20 m in a straight line, ii) forward slalom with short changes of direction, iii) forward slalom with wide changes of direction and iv) square course. The Kappa coefficient was used to test content validity, obtaining moderate to almost perfect agreement results. Construct validity was also demonstrated with very large to almost perfect correlations between tests and sessions. Good reliability was found using intra-class coefficients (>0.86), standard error of measurement (<10.8%) and Cronbach´s alpha (>0.86). The comparisons between CP profiles (i.e. sport classes) demonstrated that those with mild impairment performed faster, and those with impairment of ataxia and dyskinesia performed worse. The four tests could have applications in classification, but may also be applied by the CP football coaches to improve athlete agility and football skills.

## 1. Introduction

Football for individuals with cerebral palsy (CP football) is played by para-athletes with a minimal impairment criterion of ataxia, hypertonia, or athetosis (i.e. three impairment types that are most commonly associated with individuals having neurological impairment). Being eligible to compete in this para-sport means presenting motor control impairment of a cerebral nature, causing a permanent and verifiable activity limitation [1]. This brain injury may be due to cerebral palsy (CP), stroke, or traumatic brain injury, compromising the function of the lower limbs and constraining the performance of specific skills, such us jumping [2], sprinting [3], changing directions [4], or dribbling skills [3]. Therefore, athletes competing in CP football have an impairment that leads to a competitive disadvantage, and, consequently, requires a system that minimizes the impact of impairments on sport performance, ensuring that the success of an athlete is determined by his/her skills, fitness, power, endurance, tactical ability, and/or mental focus [5]. This system is called classification.

Classification is a defining feature of para-sport. It is defined as grouping athletes into sport classes according to how much their impairment affects fundamental activities in each specific sport or discipline, and that those classification systems should be evidence-based and sports-specific [6]. In CP football, during the last three decades (i.e. since its introduction at the 1984 Paralympic Games), a functional system was applied in this para-sport, developed by the Cerebral Palsy International Sports and Recreation Association [7]. The system comprises eight functional classes: the first four groups (classes 1–4) correspond to athletes who need a wheelchair to perform any sport, while the last four groups (classes 5–8) host ambulant athletes, that is, those eligible for CP football. Specifically, those with moderate spastic diplegia where the function of lower limbs is limited by bilateral spasticity are grouped in the sport class FT5; those with a moderate ataxic or athetoid profile involving the four limbs and trunk in the sport class FT6; those with moderate hemiplegia where one side of the body (right/left arm and leg) is affected by spasticity belong to the sport class FT7; and those with mild involvement of diplegia, ataxia/athetosis or hemiplegia, also called minimal impairment criteria to be eligible in this para-sport, are allocated in the sport class FT8 [8].

Athletes who practice football should have basic movement patterns such as running, jumping, sprinting, crouching, changing direction and rhythm [9], requiring players perform several actions that utilize strength, speed, power, agility, stability, flexibility, and endurance [10]. This intrinsic complexity and the multiple skills required in a team sport like this is a challenge when the sport is played by people with brain injury. More specifically, players with hypertonia have an abnormal increase in muscle tension and a reduced ability of the muscle to stretch, those with ataxia exhibit impaired coordination of muscle movements and those with athetosis usually exhibit unbalanced and involuntary movements due to constant changes in muscle tone and difficulty maintaining a symmetrical posture. These impairments can hamper player performance on the field, reinforcing the desirable link between impairment and activity limitation for classification purposes. In addition, football is a sport modality characterized by intermittent motor actions of short duration and high intensity, as well as moments of longer duration and lower intensity [11,12] characterized by the requirement of short races, rapid acceleration, and decelerations, turns, jumps, and changes of direction [13]. During a football match, players make changes of direction every 2 to 4 s [14] or perform between 1200 and 1400 times during a match [15]. Change of direction (COD) ability can be described as the ability to change direction while sprinting over a pre-planned course [16], so linear and change-of-direction speeds are essential qualities for athletes who play field sports, such as football [17,18]. In our para-sport, a recent study by Yanci et al. [19] demonstrated that football players with CP covered less distance at high-intensity running and sprinting, performing a smaller number of moderate- and high-intensity accelerations and decelerations, had a lower player load and performed fewer CODs in official matches as compared with conventional football players as reported in other studies. In addition, it has been demonstrated that those players with lower impairment (FT8) covered more distance at high-intensity running (13.0–18.0 km·h−1) and sprinting (>18.0 km·h−1), and they performed more accelerations, decelerations, and CODs at a high intensity in matches than did other players (i.e. FT5/6 and FT7 groups) [20]. 

Although noticeable steps have been made to describe the relationships between impairment and activity limitation in this para-sport [21], relevant questions remain to be solved for the development of a sports-specific, reliable, and evidence-based classification system in CP football. First, recent and relevant research on this topic has considered para-athletes with the brain impairments of ataxia, athetosis, or hypertonia as a unique group, so the relationships between impairment and limitation of activity would be biased. Second, the identification of the relationships between impairment and sport performance presents another particular challenge in team para-sports [22], because there are several performance factors occurring at the same time [21]. Furthermore, the inclusion of the ball when the relationships between impairment and limitation of activity are studied may also influence testing reproducibility [3].

Because football players with CP lack a repertoire of motor skills to perform the varied skills that football requires, and CODs have a prominent role during a football match, there is a need to develop a valid and reliable test to evaluate this. This study aims to explore the validity and reliability of a test battery [23] to assess CODs, together with ball dribbling in para-footballers with CP. More specifically, the study aims to validate the content validity through evaluation by a panel of experts in the field of CP football to analyze the reliability of the test battery in relation to its internal consistency (i.e., construct validity), intraclass correlation index, temporal stability, and association between the tests. In addition, the performance outcomes by players with different CP profiles (i.e., spastic diplegia, athetosis/ataxia and spastic hemiplegia) and levels of impairment (i.e., ambulant individuals with moderate vs mild CP) are also discussed. 

## 2. Materials and Methods

### 2.1. Participants

Thirty-five male football players with CP from three different countries (Brazil = 12, Spain = 10 and Portugal = 13) participated in the study. The total sample was characterized according to age (24.8 ± 6.3 year), body mass (67.5 ± 8.2 kg), height (173.3 ± 7.1 cm), and body mass index (22.5 ± 2.4 kg/m^2^), and descriptors per sport class appear in Table 1. Thirty-three para-athletes (94.3%) had a diagnosis of CP, and the remaining two para-athletes (5.7%) had traumatic brain injury and stroke, respectively. Five of the para-athletes (14.3%) acquired their brain injury in adulthood, and thirty para-athletes (85.7%) had a congenital deficiency. The inclusion criterion included the eligibility for CP football according to the IFCPF classification rulebook [1] and having a valid sports license for a national competition when the data collection took place (i.e., sports season 2017/18). As an exclusion criterion, goalkeepers did not take part in the study considering that the purpose was to evaluate movements with specific CODs during a football match. Participants were informed that participation was on a voluntary basis and they were recruited through an invitation letter that was sent to relevant sports clubs in each of the three countries. Written informed consent was obtained from participants and their trainers, and data collection was conducted in each of the three countries. The study was approved by the Institutional Review Committee of the Miguel Hernández University (reference number DPS.RRV.01.14) and complied with the recommendations of the Declaration of Helsinki.

### 2.2. Measurements

#### 2.2.1. Test #1. Ball Dribbling in a Straight Line

**Figure 1:**
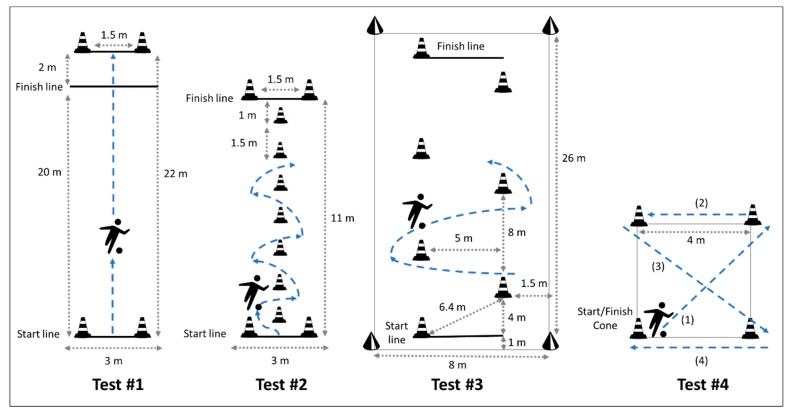
A schema for the four change of direction (COD) tests: ball dribbling in a straight line (1a, #1), slalom with short CODs (1b, #2), slalom with large CODs (1c, #3) and square (1d, #4).

In a defined area of 3 × 22 m, the athlete dribbles the ball over 20 m (Figure 1a). The athlete must run across the two cones (1–5 m apart) 2 m after the finish line, and the test is invalid in the case of the ball passing one of the lateral boundaries.

#### 2.2.2. Test #2. Ball Dribbling with Short Slalom CODs

In a defined area of 3 × 11 m, the athlete dribbles the ball between seven cones with 1.5 m separating each cone and 1 m from/to the start/finish lines (Figure 1b). The test is invalid in the case of the ball passing one of the lateral boundaries or the athlete floors a cone during the course. 

#### 2.2.3. Test #3. Ball Dribbling with Long Slalom CODs

In a defined area of 8 × 26 m, the athlete dribbles the ball between five cones with 6.4 m of separation (i.e., diagonal distance) (Figure 1c). The test is invalid in the case of the ball passing one of the lateral boundaries or the athlete floors a cone during the course. 

#### 2.2.4. Test #4. Ball Dribbling in a Square

In a defined area of 4 × 4 m, the athlete starts at the bottom left cone (Figure 1d) and (1) runs a diagonal course to the front cone, (2) performs a COD towards his left side and runs to the opposite cone, (3) changes the direction towards his right-side to run again the diagonal of the square and (4) changes the direction towards his left-side again to run towards the same cone where the test began. The test is invalid in the case of the athletes losing control of the ball or the athlete floors a cone during the course. 

All the measurements were conducted on artificial grass with an official football ball (Nike Strike 5, Oregon, USA), and three stopwatches (Casio HS-3V, Tokyo, Japan) were also required for data acquisition. All performance outcomes were recorded and expressed in seconds (s). All the athletes performed the tests properly equipped with clothing and boots suitable for practicing the sport.

### 2.3. Procedures

#### 2.3.1. Expert Consultation

Because the battery of test proposed by Martins [23] has not been previously applied to para-footballers with CP, prior to the study, an expert consultation was conducted. Four experts with deep knowledge of CP football (two national coaches, the IFCPF head of classification and one university professor with expertise in adapted physical activity and para-sports) were consulted about the content validity of the test battery. The principal investigator of the study interviewed the four experts, asking them the following four questions regarding to the four tests used in this study: i) about the understanding of the tests and its procedures with four possible responses (very easy, easy to understand, hard to understand or very hard to understand), ii) about the applicability of the tests (i.e., space and material required) with the same four possible responses, iii) whether the tests reproduce similar demands as in a CP football match with four possible responses (yes, they reproduce very well; yes, it is possible to see proximity; no, proximity is not visible; or no, it has no proximity), and iv) whether training with these tests would improve agility, COD, coordination, balance or football technique with two possible responses (yes or no). 

#### 2.3.2. COD Performance

In general, all the tests were applied under the same conditions across the different countries and groups of para-athletes. As the four COD tests aim to reproduce the movements made by the para-athletes during a football match and they must perform them in the shortest possible time, a warm-up was conducted prior to data collection sessions to increase the athletes´ preparation for subsequent efforts and maximize their performance [24]. An active warm-up was performed, consisting of 15 min of running with and without the ball and passing the ball with a goal to increase muscle temperature, blood flow and metabolic responses, allowing for better performance in each athlete [25]. All the para-athletes and their coaches received the testing protocols prior to the first testing session. 

The four tests were conducted in a single-day session, always following the same order of execution; that is, straight ball dribbling, ball dribbling in short and long slaloms and ball dribbling in the square area. The athletes were instructed to use their dominant or preferred foot for ball dribbling, but there were no restrictions for using both feet. The athlete started the test at the “Ready, set, go” commands provided by one of the observers. Every test was performed three times, having 3 min of rest between trials and a minimum of 5 min between tests [26]. Every trial was measured by three independent observers equipped with a stopwatch, and the outcome of the trial was the mean value of the three records. All the timekeepers who collaborated in each of the countries were properly instructed on handling the timers and had prior knowledge about the testing protocol and how they should act. The best trial per athlete per tests was used for data analysis.

Three days after the first test session, a retest session was performed following the same protocol. 

### 2.4. Data Analysis

The results are presented as means ± standard deviations. Regarding the content validity of the data reported by the experts, the Kappa coefficient was used with the following interpretation: 1.00–0.81 as almost perfect agreement, 0.81–0.61 as substantial, 0.41–0.60 as moderate, 0.40–0.21 as fair, 0.20–0.01 as none to slight and ≤0 as no agreement [27]. Reliability between sessions was assessed using intraclass correlation (ICC) and standard error of measurement (SEM) as metrics of relative and absolute reliability, respectively. ICC values >0.90 were considered excellent, 0.75–0.90 as good, and < 0.75 as poor to moderate [28]. The SEM was calculated using the following formula: $SEM=SD\cdot \;\sqrt{1-ICC}$ [2]. Test reliability was also assessed using Cronbach’s alpha, considering scores over 0.70 acceptable [29]. A t-test of related measurements was used to calculate the differences between the scores obtained in the two testing sessions.

The relationships between the different tests were assessed using Pearson’s product-moment correlation (r), with the following scale of magnitudes to evaluate correlation coefficients: <0.1, trivial; 0.1–0.3, small; <0.3–0.5, moderate; <0.5–0.7, large; <0.7–0.9, very large; and <0.9–1.0, almost perfect [30]. A one-way analysis of variance (ANOVA) with the least significant difference post hoc comparison (Tukey´s correction) was used to examine the mean differences among sport classes. Practical significance (i.e. effect sizes) was calculated using Cohen’s d with Hedges´ correction (*d*_g_) for (sub)samples below 20 participants [31]. The interpretation of this effect size was made according to the following values: <0.25 = trivial; 0.25–0.50 = small; 0.50–0.80 = moderate; and >0.80 = large [32]. Statistical significance was set at *p* <0.05. Data analysis was performed using the Statistical Package for Social Sciences (SPSS Inc, version 24.0 for Windows, Chicago, IL, USA).

## 3. Results

### 3.1. Content Validity

The four experts who were consulted on the feasibility of applying the test battery in a group of para-footballers with CP reported that tests were very easy (75%, *n* = 3) to easy (25%, *n* =1) to understand (κ = 1.00, *p* = 0.046). The level of agreement about the applicability of the test in this population was perfect (100%, κ = 1.00, *p* < 0.001). With regard to the reproducibility of a CP football match throughout the test’s battery, all the experts agreed “yes”, but 50% (*n* = 2) reported “they reproduce very well” and the other 50% reported that “it is possible to see proximity” (κ = 1.00, *p* = 0.046). With regard to whether the test battery could improve COD, agility, coordination and balance, 75% of the experts responded “yes” and 25% responded “no” (κ = 0.43, *p* = 0.046). 

### 3.2. Test Reliability and Reproducibility

Table 2 shows the scores obtained in testing sessions one and two (i.e., 72 h retest). Considering the mean values of the best trials in the two sessions, the test (#1) where ball dribbling was only required in a straight line was the test with the lowest scores (4.68 ± 1.25 s), followed by test #2 with short slaloms (7.87 ± 2.29 s) and test #4 with a square course (10.34 ± 2.46 s), while test #3 with demanding wide slaloms was the test that required more time for its completion (19.61 ± 4.78 s). All the tests exhibited good-to-excellent relative reliability (ICC = 0.86–0.97), with percentages of SEM (i.e., absolute reliability) from 4.6% (test #4) to 10.8% (test #2). However, the performance between testing sessions significantly improved for all the tests (*p* < 0.05). 

### 3.3. Construct Validity

To assess the construct validity of the test, that is, if they measure similar football skills, Pearson’s correlations were conducted between all the tests. Due to significant differences being obtained between sessions 1 and 2, the correlations were done considering the tests scores in each individual session (Table 3). The correlations between sessions for each individual test exhibit very large to almost perfect correlations (*r* = 0.88–0.97; *p <* 0.001). Among the tests, similar correlations were also obtained for session 1 (*r* = 0.85–0.94; *p <* 0.001) and session 2 (*r* = 0.72–0.93; *p <* 0.001). 

### 3.4. Between-Group Differences

To assess between-group differences, the scores of session 2 were used because better performance scores were obtained in that session. The ANOVA only showed overall between-group differences in test #2, including the pair comparison of FT6 (i.e., moderate ataxia/athetosis) vs FT8 (i.e., minimal impairment criteria) sport classes. However, Table 4 shows that para-athletes with athetosis and ataxia (i.e., FT6) are those with the worst performance in all the tests, while those with minimal impairment criteria (i.e., FT8) obtained the best scores. In addition, para-footballers belonging to class FT8 had large effect sizes when compared with the other sport classes in all the tests (*d*_g_ = 0.82–1.75, large). 

## 4. Discussion

The aim of this study was to evaluate the content and construct validity, and the reliability of four agility tests requiring ball dribbling in football players with CP. The previous research in this population demanding sport-specific skills is scarce [3], so this study provides new evidence about the feasibility of its implementation for classification or training purposes. Apart from this, this is the first time that the test battery [23] has been applied to para-footballers with CP after an expert consultation verifying that the test battery was feasible and pertinent for this population. Therefore, a new group of tests to assess CODs in para-footballers with CP is now available with good reliability (α = 0.86–0.97), increasing the recent evidence for the Illinois Agility Test (IAT) or the Stop and Go (S & G) test [3], unique evidence on this topic to the best of the authors knowledge. The study by Reina et al. [3] investigated the effect of the ball dribbling when performing the abovementioned agility test and a 40 m straight sprint, with and without ball dribbling. This cross-sectional study involved 82 international para-footballers with CP, but the measurements were done in a single session. As a result, the between-session reliability was not explored (as our study did). The IAT with ball dribbling had an ICC value of 0.84 and an SEM of 6.3%, while the S&G test had an ICC of 0.48 and an SEM of 9.8%. The three tests of this study requiring CODs (#2, #3 and #4) had better ICC and SEM values, except the SEM of test #2 (SEM = 10.8%). 

Although test #1 does not really demand a COD due to it consisting of a straight-line sprint with ball dribbling, this study reports its reliability when ball dribbling is required in a shorter run as compared with the study by Reina et al. [3], where para-athletes had to run 40 m with partial measurements at 10 m and 25 m. Our study exhibits better ICC values (0.95 vs 0.84 at 10 m, 0.76 at 25 m and 0.73 at 40 m) and similar SEM values (6.3% vs 4.5% at 10 m, 6.2% at 25 m, and 6.5% at 40 m). The 20 m sprint has been recently used to explore the relationships between performance and match load using GPS technology [21], but without involving ball skills during the test. Therefore, this study provides new evidence about the feasibility and reliability for implementing a sport-specific test that better replicates the demands of the real game [19,20,21]. 

Test reproducibility deserves a special mention, as significant differences were found between test and retest sessions. This is the first study reporting between-sessions reliability in para-athletes with CP, suggesting the potential bias of a learning effect. The IPC Position Stand on Classification in Paralympic Sport [5] identifies and describes several criteria for a valid measure of impairment: measures should be objective, reliable, precise, specific to the impairment of interest, parsimonious (i.e. account for the greatest possible variance in sports performance) and, as much as possible, be resistant to the effects of training (i.e. when athletes undertake rigorous, sport-specific training, pre-training measures of impairment should not be significantly different from post-training measures of impairment) [33]. Although our tests assess performance when sprint/CODs with ball dribbling are required and not the impairment, the improvement in performance after 72 h from the first session is a remarkable finding for classification purposes. Current methods of classification include both novel motor tasks (i.e. tasks that are unlikely to have been practiced by the athlete in the usual course of training for his or her sport) and sport-specific activities (i.e., tasks that are likely to have been frequently practiced by athletes training for the sport), so a familiarization with the classification protocols would be recommended to avoid bias in the decision-making of classifiers, that is, the para-athlete exhibits more limitation of activity than really he/she has due to unfamiliarity with the tests. 

The construct validity of the test battery has also been demonstrated, with very large to almost perfect correlations found between tests and testing sessions (*r* = 0.72–0.97), so the tests measure similar components of agility. These correlations are higher than those obtained in other studies with this population, correlating the IAT with the S&G test (*r* = 0.35, *p* < 0.01) and the straight sprint at 10 m (*r* = 0.35, *p* < 0.01), 25 m (*r* = 0.50, *p* < 0.01), and 40 m (*r* = 0.34, *p* < 0.01) [3]. The correlations in this study are also higher than those obtained in a study with 99 international para-footballers with CP [4], where the relationship between the IAT (without ball dribbling) and the Modified Agility Test was explored. Whereas the study by Reina et al. [3] demonstrates a higher variability in sports performance when agility tests performed while ball dribbling is required, our study increases the options for testing agility in para-footballers with CP with shorter courses and time requirements for its completion, especially in tests #2 and #4. 

Regarding the between-group comparisons, our results should be interpreted with caution due to the number of players per sport class. It should be mentioned that the sport class distributions obey the para-sport rules, where two players of the sport classes FT5/FT6 must be on the field of play and no more than one FT8 player would be included in the line-up. Nevertheless, the large effect sizes when comparing FT8 para-footballers with the other three sport classes demonstrated that those with a lower level of impairment exhibit better sports performance [2,3,4,8,19,21,34]. In addition, it was found that FT6 players performed the worst in all the tests, reinforcing the idea that those with dyskinesia or ataxia would be those para-athletes in which brain injury may affect, to a higher extent, sports performance (i.e., limitation of activity) when multiple skills are required. Para-athletes who have ataxia (involuntary movements and impaired coordination), athetosis (involuntary contractions of the muscles) or dystonia (repetitive torsions and movements or abnormal postures) are individuals that have general problems with balance and performing departures, stops and turns during a race [35]. Accordingly, a recent study conducting a biomechanical comparison of the initial sprint acceleration performance and technique in an elite athlete with CP of this profile and able-bodied sprinters demonstrated the impact of this type of CP on performance in a competition-specific acceleration movement [36]. Therefore, when the ball is included during testing, driving it makes it more difficult to accomplish the task, constraining their motor control, and they take longer than the other sport classes.

FT5 players have been considered the other “low sport class” sub-group (i.e., more impaired for the real game) [21], and they were the second sub-group with lower performance (i.e., after the FT6 sport class), except for tests #1 and #2, with scores similar to FT7 para-footballers. Athletes of class FT5 are individuals with spastic diplegia, that is, they present with spasticity in their legs, hips, and pelvis. This high abnormal muscle tone causes impairment in the voluntary and passive movements of the legs, rotational ankle abnormalities followed by poor pelvic alignment [37]. These compromises cause difficulty and reduced steps for athletes to move around, consequently making them take longer to make a COD. On the other hand, those in class FT7 are para-athletes with spastic hemiplegia, so it is common to observe limitations in gait, increased limb asymmetry or reduced stride length [38]. Therefore, knee and hip control are also affected by spasticity and possible loss of range of motion due to muscle contraction. However, the similar results in tests #1 and #2 (i.e. the two tests with no or shorter CODs) would be explained by the compensatory strategies that para-footballers may use. That is, those with spastic diplegia have shorter strides but may use both feet for better ball control, while those with spastic hemiplegia usually use the dominant or unimpaired foot for ball control. The differences between these two sub-groups slightly increased in tests #3 and #4, where CODs are more remarkable, so those with spastic hemiplegia can use the unimpaired leg for better stabilization when pivoting to perform the required COD.

Some limitations should be mentioned. First, the number of participants per sport class constrains the between-group comparisons. However, the study has been applied with participants from three different countries, which have national teams participating at an international level. Second, not all the participants had international experience, or they could not be considered well-trained para-athletes, being a potential factor that explains the differences in performance between testing sessions. However, classification is a process that is applied at local, regional, and international stages, so the level of training or the familiarization with the tests must be considered in further research. 

## 5. Conclusions

The results of this study show good values of reliability and validity for all tests where ball control is required: ball dribbling in a straight line (#1), forward slalom with short CODs (#2), forward slalom with wide CODs (#3), and square course (#4). Tests #3 and #4 show the best reliability results, and they would be recommended to assess agility and COD in para-footballers with CP. In addition, the four tests may also help CP football coaches to monitor their player's performance, which will help the physical, tactical, and technical conditioning of players.

## Figures and Tables

**Table 1:** Characteristics of the para-footballers with CP.

	Bilateral Spasticity or Diplegia	Dyskinesia or Ataxia	Unilateral Spasticity or Hemiplegia	Mild Impairment
Sport Class	FT5	FT6	FT7	FT8
*N*	5	8	19	3
Age (years)	21.6 ± 2.8	24.5 ± 4.0	25.2 ± 7.3	28.0 ± 11.1
Height (cm)	175.3 ± 7.3	170.6 ± 8.1	175.8 ± 7.8	172.9 ± 8.7
Body Mass (kg)	67.4 ± 9.5	60.6 ± 5.3	69.7 ± 12.9	72.3 ± 5.0
BMI (kg/m^2^)	21.8 ± 2.2	21.0 ± 1.7	22.8 ± 3.3	24.4 ± 2.4

*N* = number of participants, cm = centimeters, kg = kilograms, BMI = body mass index, M ± SD.

**Table 2:** Means and standard deviations for tests and sessions and reliability scores.

	Session 1	Session 2	ICC_1–2_	SEM (%)	IC (95%)	α	t	*p*
Test #1 (s)	4.8 ± 1.3	4.6 ± 1.2	0.95	0.29 (6.3)	0.91–0.97	0.95	3.03	0.005
Test #2 (s)	8.3 ± 2.5	7.5 ± 2.0	0.86	0.85 (10.8)	0.73–0.92	0.86	3.56	0.001
Test #3 (s)	20.0 ± 5.0	19.2 ± 4.6	0.96	0.98 (5.0)	0.93–0.98	0.96	3.20	0.003
Test #4 (s)	10.5 ± 2.6	10.2 ± 2.4	0.97	0.47 (4.6)	0.94–0.98	0.97	2.32	0.026

s = seconds, ICC = intra-class coefficient, SEM = standard error of measurement, IC = interval of confidence, M ± SD.

**Table 3:** Correlations between tests and sessions.

	Test #1 s2	Test #2 s1	Test #2 s2	Test #3 s1	Test #3 s2	Test #4 s1	Test #4 s2
Test #1 s1	0.950**	0.846**	0.780**	0.942**	0.928**	0.898**	0.885**
Test #1 s2		0.766**	0.715**	0.894**	0.882**	0.832**	0.815**
Test #2 s1			0.877**	0.887**	0.925**	0.919**	0.902**
Test #2 s2				0.813**	0.854**	0.815**	0.852**
Test #3 s1					0.963**	0.942**	0.937**
Test #3 s2						0.954**	0.934**
Test #4 s1							0.966**

s = session, ** *p <* 0.001.

**Table 4:** Between-group comparisons according to para-athlete sport class.

Test	Class	M ± SD	F	*p*	Pair Comparisons (*d*_g_)			
					FT5	FT6	FT7	FT8
#1	FT5	4.5 ± 0.9	1.46	0.246	--	−0.50	--	0.99
	FT6	5.2 ± 1.5				--	0.55	1.08
	FT7	4.5 ± 1.1					--	0.82
	FT8	3.6 ± 0.5						--
#2	FT5	7.5 ± 2.6	3.31	0.033	--	−0.49	0.11	1.02
	FT6	8.8 ± 2.4				--	0.83	1.64 *
	FT7	7.3 ± 1.4					--	1.66
	FT8	5.0 ± 0.1						--
#3	FT5	20.6 ± 5.1	1.58	0.214	--	−0.11	0.36	1.15
	FT6	21.1 ± 4.0				--	0.50	1.58
	FT7	18.8 ± 4.7					--	0.82
	FT8	15.0 ± 1.3						--
#4	FT5	10.8 ± 2.9	1.63	0.202	--	−0.12	0.27	1.13
	FT6	11.1 ± 2.0				--	0.42	1.75
	FT7	10.1 ± 2.4					--	1.01
	FT8	7.7 ± 0.4						--

FT = sport class, *d*_g_ = effect size, * *p <* 0.05.
